# Dissecting the impact of heat stress on heat-shock response and skin microbiota in farmed fish in a recirculating aquaculture system in Singapore

**DOI:** 10.1128/spectrum.00568-24

**Published:** 2025-08-13

**Authors:** Tze Hann Ng, Sobana M, Xian Zhe Chew, Thiviya Nair, Jing Wen Chow, Adrian Low, Henning Seedorf, Giana Bastos Gomes

**Affiliations:** 1 Temasek Life Sciences Laboratory, National University of Singapore, Singapore, Singapore; 2 James Cook University in Singapore, Singapore, Singapore; 3 Department of Biological Sciences, National University of Singapore, Singapore, Singapore; Panepistemio Thessalias Tmema Geoponias Ichthyologias kai Ydatinou Periballontos, Volos, Greece

**Keywords:** skin microbiota, heat-shock response, heat treatment, *Lates calcarifer*

## Abstract

Periodic heat treatment is a method used by aquaculture to reduce fish-borne pathogens. Few studies have examined the effects of heat treatment on fish gene expression and changes to skin microbiota, despite their role in fish health. Thus, this study aimed to evaluate the fish heat-shock response and changes to the skin microbiota during heat treatment in a commercial fish farm with a recirculating aquaculture system. Differences in host gene expression were determined by quantitative PCR (qPCR), and we found that heat treatment increased heat-shock protein (*hsp70* and *hsp90*) and cytokine (interleukin-1-beta and *tumor necrosis factor-alpha*) transcript expression. The 16S ribosomal RNA gene amplicon analysis of fish skin microbiota and qPCR detection of scale drop disease virus (SDDV) from skin revealed a reduction in opportunistic bacteria and viruses of surviving fish after heat treatment. However, after heat treatment, moribund fish exhibited the highest levels of heat-shock responses and opportunistic pathogen abundance. We inferred that heat treatment also induced an overreaction to temperature stress causing dysbiosis of fish microbiota and even death. We also propose the value of the skin microbiota Pseudomonadota/Bacteroidota ratio as a biomarker for aquaculture fish health.

**IMPORTANCE:**

The application of disruptive technologies such as recirculating aquaculture systems (RAS) to aquaculture aims to increase productivity and maximize economic yield. However, significant challenges remain in pathogen and parasite prevention despite RAS. Developing practical solutions for producing healthy juveniles in nursery systems will make profound contributions to sustainable aquaculture. In this study, we used an unconventional strategy, exposing juveniles to the pathobiome in the environment, followed by water heat treatments to enhance fish responses. We found that short-term water temperature fluctuations have no impact on skin microbiota, whereas induced heat-shock responses reduced opportunistic bacteria and viruses in surviving fish. We inferred that manipulating microbial-host and environmental interactions, together with the enhanced functional capacity of fish stress/immune response, could have an impact on disease control in aquaculture. We also determined the use of skin microbiota in fish health monitoring.

## INTRODUCTION

Commensal and opportunistic bacteria in fish skin mucus comprise part of the fish skin microbiota (1). Fish’s primary immune response is extremely active in mucus (2, 3), protecting against environmental pathogens. However, many pathogenic and opportunistic bacteria can adhere to skin mucus, evade the fish defense barrier, and promote infection. Overgrowth of these bacteria can disrupt the skin microbiota homeostasis, thus leading to dysbiosis. However, not all skin microbial dysbiosis causes disease (4, 5). The assessment of skin microbiota in fish species has received relatively little attention (as compared to that of fish gut microbiota, which has been analyzed for more than 145 species (6), but there is significant and growing interest in understanding more about this exciting topic, especially for farmed fish (1, 7, 8). Skin microbiota of fish is strongly linked to their surroundings and influenced by many factors including fish species, geographic origin, environmental dependent factors, and stressors (7, 9–11). Early research demonstrated seasonal changes in skin bacterial abundances in North Sea cod (*Gadus morhua*), with Achromobacter and luminous strains appearing more frequently in the winter and Pseudomonas strains increasing in the summer (9). The dynamics of the skin microbiota were found to be related not only to temperature changes but also to seasonal plankton outbursts (9, 10). Factors such as high stocking density and hypoxia are also common stressors that cause shifts in the commensal microbiota, especially in aquaculture production (12). The microbiota of unstressed fish is dominated by taxa with probiotic and antibacterial properties, whereas the microbiota of fish under hypoxia seems to be dominated by potential pathogens. The crowded conditions and low oxygen present in some fish farms can stress fish and can cause dysbiosis in the skin microbiota, which promotes the growth of opportunistic pathogens (12–15).

Water temperature is another factor that disrupts the composition and diversity of the gut and skin microbiota of healthy fish (15). The equilibrium of skin and gut microbiota was disturbed by temperature fluctuations in chum salmon, especially Vibrio in fecal samples (15). Vibrio is present in water and possibly in low numbers in the skin and gills of apparently healthy fish (16, 17). However, many Vibrio species are common opportunistic pathogens, particularly in controlled and intensive aquaculture systems (18–20). Higher water temperature at the normal host range can increase the expression of virulence genes that can promote infection and disease outbreaks (21, 22). Fish optimal growth temperatures are usually favorable for the growth of these pathogenic bacteria during infection processes. However, temperatures higher or lower than the normal host range may limit pathogenic virulence in fish, as has been observed with rhabdoviruses, betanodaviruses, Yersinia, Flavobacterium, Lactococcus, and Vibrio species (21, 23). This is due to temperature changes that can have combinatorial effects on fish stress response, immune system, and metabolic rates (24, 25).

Heat-shock proteins, including HSP70 and HSP90, are some of the main responsive factors during heat treatment, with either downregulated or upregulated expression profiles when exposed to temperature changes (26, 27). In a study of carp (*Catla catla*), fishes were exposed to six different temperatures from 10 °C–35 °C. It was found that fish exposed to temperatures below 25°C were most impacted in their food intake and ability to digest food and by a suppressed immune system. Although *hsp70* was mostly responsive to rises in temperature, *hsp 60* was sensitive to temperature drops (24). Not only in fish but water heat treatment at 37°C for 30 minutes also increased resistance against Vibrio species in Artemia, which was linked to the induction of heat-shock protein genes (28, 29).

The effect of cold temperature changes on fish immunity has been well studied, but the potential effects of a rapid shift to high temperature on fish heat-shock responses and immunity, as well as their ability to mount protective responses against infections, have only recently begun to be investigated (30). Even though water heat treatment is used as a disease management practice in farmed fish (particularly around Asia) aiming to eliminate pathogens (especially viruses) and prevent disease outbreaks, little research has been done to understand how environmental stress, microbial communities, and host physiology interact to improve fish functional capacity following heat treatment. To study such interactions, we collected samples from a commercial farm in Singapore that raises barramundi (*Lates calcarifer*). We investigated how fish heat-shock responses and skin microbiota vary as water temperature increases to 37°C–39°C (common farm practice) from tropical temperatures of 29 to 31°C on the farm. 16S rRNA gene amplicon analyzes were conducted on fish fins and kidney samples. Protective impacts were assessed by the fish appearance (survive or moribund after heat treatment), pathogen detections in fin and kidney, and heat-shock responses in the liver. Insights from this study could reveal key biomarkers for use in disease surveillance and fish health monitoring in aquaculture.

## MATERIALS AND METHODS

### Fish rearing system and experimental design

**Fig 1:**
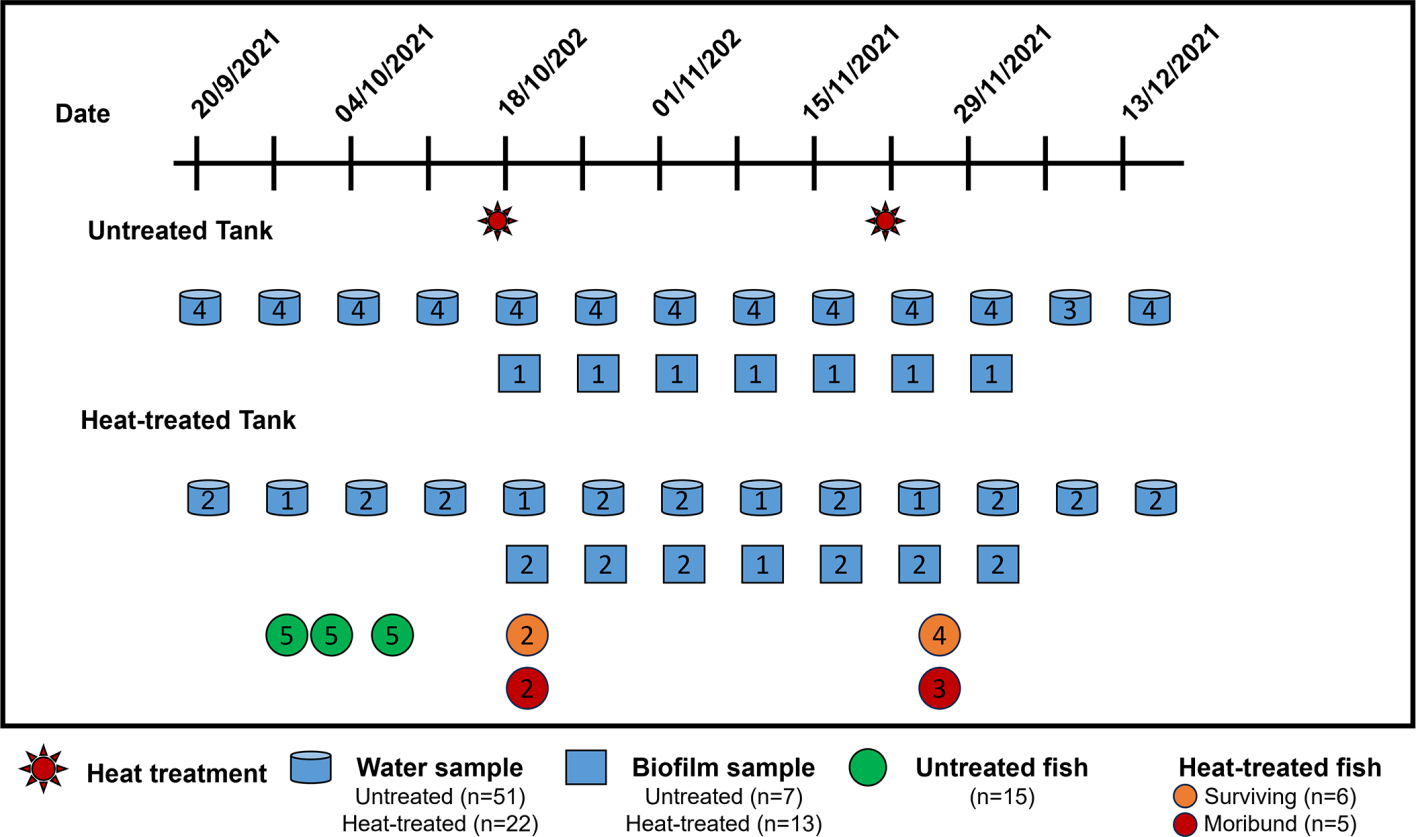
An illustration scheme of experimental design and sampling. Heat treatment was performed once per day for 3 consecutive days to complete the treatment course. The numbers labeled in each shape represent the sampling count per treatment on the corresponding sampling day.

To study the suitability of heat treatment practices for disease management, we collaborated with a commercial fish farm in Singapore that uses a recirculating aquaculture system. This farm raises barramundi in tanks (tank size; diameter: 8.45 meters, height: 2.55 meters, volume: 143 m^3^) with a capacity of up to 12 tons of fish per tank. Fish were kept at tropical ambient temperatures (range 29 to 31°C) on the farm. The study included two heat-treated tanks and four untreated tanks. The heating cycle was performed in the same tanks which were never emptied during the study period. Heat treatment raised the water temperature in heat-treated tanks to 37°C–39°C and held for 1 hour before gradually returning to 29°C–31°C by adding fresh seawater. This heat treatment was performed once per day for 3 days to complete the treatment course. Figure 1 shows an illustration of experimental design and sampling. During the sample period from 20 September 2021 to 13 December 2021, mortality was observed not only after heat treatment but also during routine fish transfers in and out of tanks, which are regular operations that may introduce pathogens and increase mortality.

Fish samples were collected from one of the heat-treated tanks, including both surviving (*n* = 6) and moribund (*n* = 5) fish on two heat-treatment days, while untreated (*n* = 15) fish were collected on three non-heat treatment days. Moribund fish exhibited darker skin, lethargy, abnormal swimming behavior, and gasping at the surface but no schooling behavior. The fish were immediately euthanized with Aqui-S (40 mg/L; AQUI-S, New Zealand), their length and weight recorded, and then stored on ice. The sampled fish varied in size from 12.5 to 20.7 cm. Each fish’s liver, kidney, and fin were sampled and stored at −80°C in 95% ethanol until use. Water and biofilm samples were collected from all six tanks. Two liters of water were sampled from each tank and transported on ice to the Temasek Life Sciences Laboratory in Singapore for processing. Water samples were filtered using a filter holder set designed for 47 mm diameter filters and attached to a vacuum pump (R-300, Biofrontier Technology PTE LTD). The filter holder set was disinfected with bleach and rinsed with sterile water between each use. Water samples were prefiltered through 3.0 µm pore-size cellulose ester single-use filter membranes (Merck, USA) to remove large cells and particulate matter. The flowthrough was filtered through 0.22 µm cellulose ester single-use filter membranes (Whatman, Germany) to recover microbial cells. Cell-concentrated membranes and swabs of biofilm from the tank wall were put into 2.0 mL Eppendorf tubes and stored at −20°C until further processing. Some water and biofilm samples could not be collected due to practical and safety issues at the commercial farm, especially in heat treatment tanks.

### Microbiological compositions by 16S rRNA gene-based sequencing

#### DNA extraction and 16S rRNA gene amplicon sequencing

The DNeasy Blood & Tissue Kits (Qiagen) were used to extract 52 tissues (26 fin and 26 kidney) from 26 fish (six surviving, five moribund, and 15 untreated) following the manufacturer’s instructions, including pretreatment for gram-positive bacteria with enzymatic lysis buffer (20 mM Tris-HCl, pH 8.0; 2 mM sodium EDTA; 1.2% Triton X-100; 20 mg/mL lysozyme) at 37°C 1 hour and proteinase K digestion for 3 hours. Whereas 73 water and 20 biofilm samples were extracted using a cetyltrimethylammonium bromide/chloroform-isoamyl alcohol-based protocol (31). PCR amplification of V4-V5 regions of the 16S rRNA gene was done using specific primers linked to barcodes (primer 515F: GTGCCAGCMGCCGCGGTAA [32]; 907R: CCGTCAATTCCTTTGAGTTT) (33). PCR products were confirmed using 2% agarose gel electrophoresis. The library contained (*n* = 145) barcoded samples and DNA was quantified using a Qubit and bioanalyzer for size distribution identification and quantification. Equal amounts of PCR products from each sample were pooled and sent to an external vendor (NovogeneAIT) for 2 × 250 bp paired-end sequencing on an Illumina NovaSeq platform (Illumina, USA). Ten kidney samples failed the sample QC and were removed from further library construction.

#### Data processing

Demultiplexed reads for 135 samples provided by the vendor were processed using the pipelines in QIIME2 Version 2021.4 (34). Single-end reads were denoised using the “qiime dada2 denoise-single” command for DADA2 and analyzed for chimera (35). Taxonomic assignment was performed using the “qiime feature-classifier classify-sklearn” command against a SILVA pre-trained classifier (silva-138-99-nb-classifier). Chloroplast, mitochondria, unidentified sequences, and Amplicon Sequence Variants (ASVs) observed with fewer than 15 reads or in less than three samples were removed. Alpha diversity (Shannon diversity) and beta-diversity (Bray-Curtis dissimilarity) were calculated in Phyloseq (36) using an even depth across samples (rarefaction depth of 90% of the minimum sample depth, in this study 34, 826 was chosen). ASV tables grouped at phylum and genus levels were used for further analysis of taxa with a relative abundance >1%. The top 10 taxa were sorted by abundance based on total abundance across all samples using the top_taxa function (R package “microbiome” [37]). Statistically significant variation in microbiota composition and diversity was detected by analysis of similarity, permutational multivariate analysis of variance (PERMANOVA), and pairwise Wilcoxon tests (38).

### Host genes quantification

#### RNA extraction and qPCR

Total RNA of liver samples from the same 26 fish [surviving (*n* = 6), moribund (*n* = 5), and untreated (*n* = 15)] was extracted using the RNeasy Plus Micro Kit (Qiagen) and Invitrogen SuperScript IV First-Strand Synthesis Kit (Invitrogen) was used to synthesize complementary DNA (cDNA) according to the manufacturer’s instructions. Based on published sequences, primer sequences for *hsp70*, *hsp90*, il1-beta, tnf-alpha, and *elongation factor 1-alpha* (ef1-alpha) genes were synthesized (Table 1). Quantitative polymerase chain reaction (qPCR) was performed using a CFX96 (Bio-Rad). Reactions were carried out in a 20 µL mixture of 1 µL diluted cDNA, 10 µL SYBR green master, 0.4 µL forward primer, 0.4 µL reverse primer, and 8.2 µL water. The qPCR program included an initial denaturation at 95°C for 3 minutes, followed by 40 cycles of denaturation at 95°C for 3 seconds, and annealing/amplification at the appropriate melting temperature for 20 seconds. Relative mRNA expression for target genes in each sample was calculated using the 2^−ΔCt^ method, and ef1-alpha was used as an internal control gene for data normalization (39). R version 4.2.2 and RStudio (40) were used for statistical analyzes (R package “rstatix” [41]) and graph generation [R package “ggplot2” (42)]. All data were shown as the interquartile range (IQR) and Kruskal-Wallis with Dunn’s *post hoc* tests was used to compare means. The Benjamini-Hochberg (BH) (43) method was used to adjust the *P*-value for multiple hypothesis tests.

### Specific pathogens quantification

#### DNA extraction and qPCR

The same DNA samples used for 16S rRNA gene-based sequencing were screened for SDDV) and *Vibrio harveyi* using specific primers, respectively. Primer sequences and probes used in this study are shown in Table 1. qPCR was performed using a Bio-Rad CFX96 (modified from Kiat et al. [44]). Reactions were carried out in a 20 µL mixture of 2 µL DNA, 10 µL probes mastermix, 0.4 µL forward primer, 0.4 µL reverse primer, and 8.2 µL water. The qPCR program with an initiation at 95°C for 3 minutes, followed by denaturation at 95°C for 3 seconds, and then 40 cycles of annealing/amplification at the appropriate melting temperature for 20 seconds. R version 4.2.2 and RStudio (40) were used for statistical analyzes (R package “rstatix” [42]) and graph generation (R package “ggplot2” [42]). All data were shown as the IQR and Kruskal-Wallis with Dunn’s *post hoc* tests was used to compare means. The BH (43) method was used to adjust the *P*-value for multiple hypothesis tests.

**TABLE 1:** Primers used in this study

Gene	Primer sequence (5′-3′)	References
*hsp70*	F: AAGGCAGAGGATGATGTC R: TGCAGTCTGGTTCTTGTC	(45)
*hsp90*	F: ACCTCCCTCACAGAATACC R: CTCTTGCCATCAAACTCC	(45)
*il1-beta*	F: CCTGTCGCATTTCAGTACGG R: ATTTCCACCGGCTTGTTGTC	(46)
*tnf-alpha*	F: GCCATCTATCTGGGTGCAGT R: AAAGTGCAAACACCCCAAAG	(47)
*ef1-alpha*	F: GTTGCCTTTGTCCCCATCTC R: CTTCCAGCAGTGTGGTTCCA	(45)

Pathogen	Primer sequence (5′-3′)	References
*V. harveyi*	F: TGAAGCTGTGATCAACTACG R: CCTGACAGTGGCTCTTGTAA	(48)
SDDV	F: AGCTGCATCGCCCATCA R: TGAGCTTTTGGAGGCATTGTAA Probe: TGCCAGTGGTACAAAC -FAM	(44)

## RESULTS

### Sequence data

A total of 135 samples were sequenced, including 26 barramundi fin, 16 barramundi kidney, 73 water, and 20 tank biofilm samples, yielding approximately 26 million raw reads. The number of reads per sample ranged from 38,696 to 239,079. These sequences corresponded to 5,355 unique ASVs and, after filtering and removing ASVs belonging to organelles, resulted in 4,802 unique ASVs for downstream analysis.

### Comparison of the environment microbial communities to fish tissues microbiota

**Fig 2:**
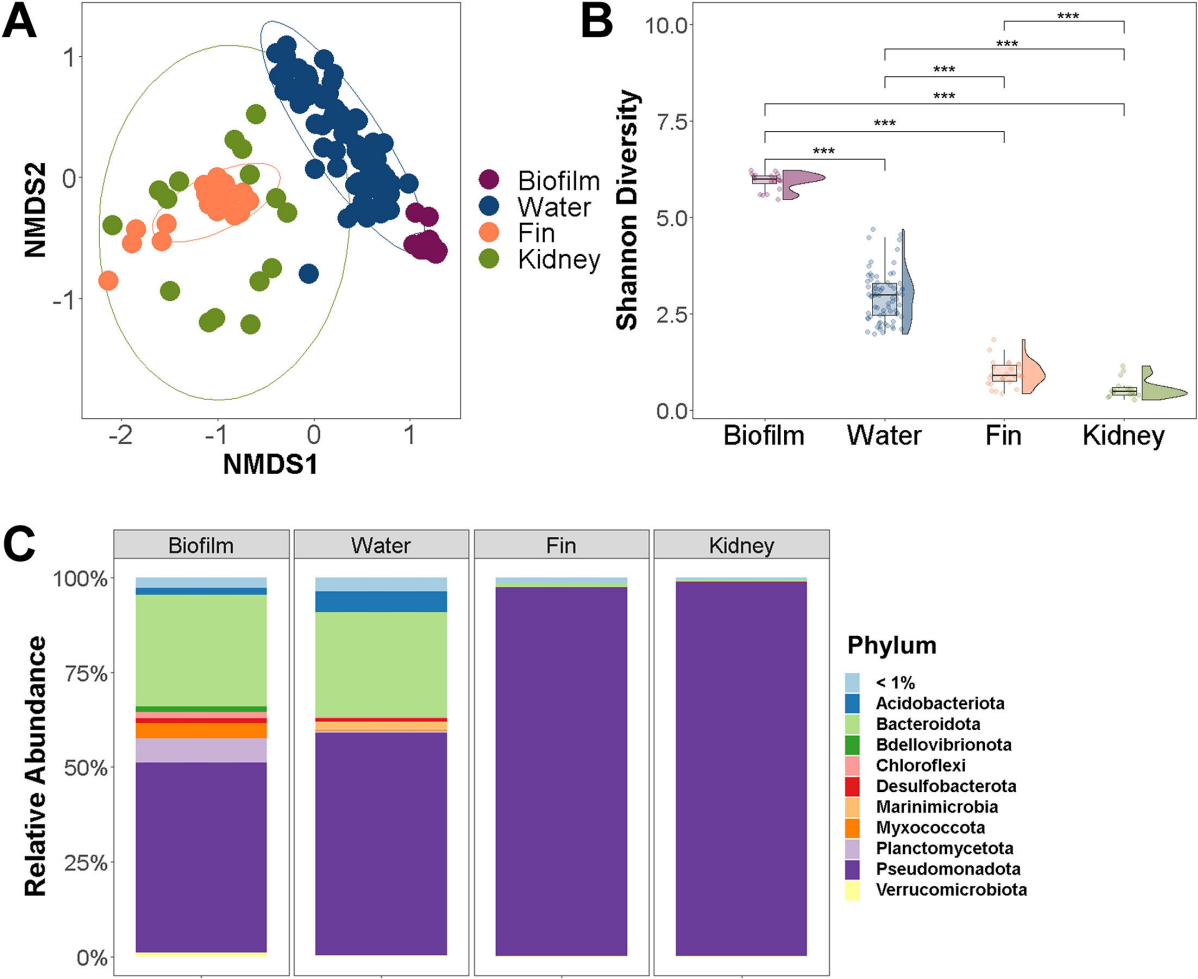
Comparison of environmental and fish tissue microbiota. (**A**) Bacterial profiles were compared by nMDS using the Bray-Curtis distance metric displaying the community dissimilarities (based on ASVs) among sample types. PERMANOVA statistic *R*²= 0.7239, *P* = 0.001. (**B**) Diversity plot of bacterial diversity comparing sample types based on Shannon index. Differences between sample types were tested (***P < 0.005). (**C**) Relative abundance of bacterial phyla among sample types. Rare taxa in each group are indicated as <1%.

We analyzed the microbial communities of barramundi tissues, water, and tank biofilm and compared the alpha diversity, beta-diversity, and compositional differences between fish and the environment. The microbiota of fish tissues (fin and kidney) differed from those of the environment (tank water and biofilm), which formed well-separated clusters with no overlap, based on non-metric multidimensional scaling (nMDS) ordination analysis with Bray-Curtis distance (Fig. 2A). The groupings were statistically significant as supported by PERMANOVA tests (*R*²= 0.7239, *P* = 0.001). Biofilm and water microbiotas had higher Shannon diversity values compared to fish tissue microbiota (Kruskal-Wallis test, *P* = 2.2 × 10^−16^; Fig. 2B). Biofilm microbiota showed significantly higher Shannon diversity compared to water communities (*P* < 0.005; PERMANOVA). In contrast, kidney microbiota had substantially lower Shannon diversity than fin microbiota (*P* < 0.005; PERMANOVA).

Compositionally, Bacteroidota (formerly Bacteroidetes) and Pseudomonadota (formerly Proteobacteria) were prevalent in the environmental samples, whereas Pseudomonadota dominated the microbiota of fish tissues (Fig. 2C). The phyla Bdellovibrionota, Chloroflexota, and Myxococcota were detected only in tank biofilms. Furthermore, Bacillota (formerly Firmicutes), which are typically prevalent in fish gut microbiota (16, 49), constituted a minor fraction (<1%) in the fin microbiota.

### Effect of periodic heat treatment on microbiota of fish tissues

**Fig 3:**
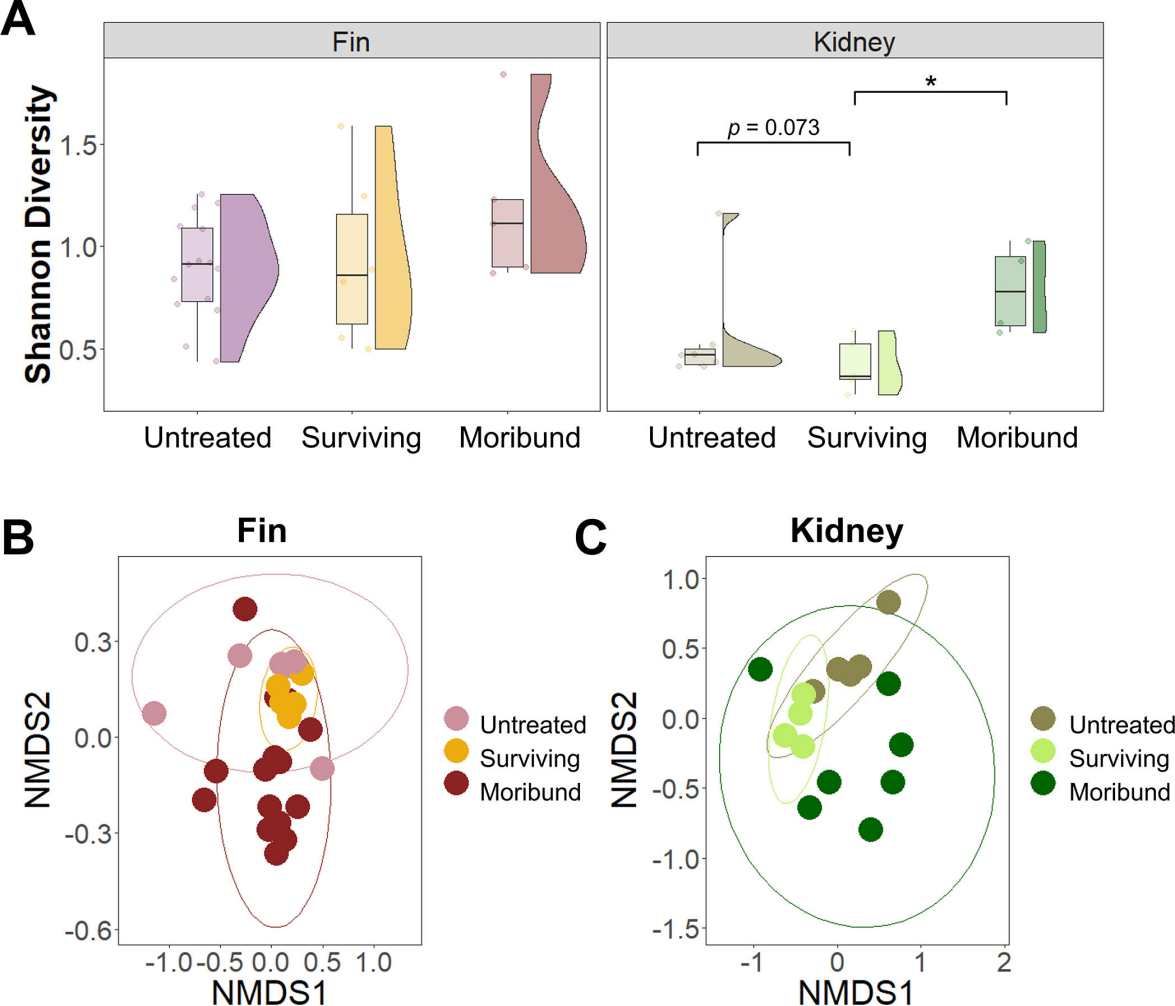
Diversity of fin and kidney microbiota in untreated, surviving, and moribund fish in a heat-treated tank. (**A**) Diversity plot of bacterial diversity comparing untreated, surviving, and moribund fish based on Shannon index. Bacterial profiles of (**B**) fin and (**C**) kidney were compared by nMDS using the Bray-Curtis distance metric displaying the community dissimilarities (based on ASVs) among fish tissue samples.

To determine the effects of heat treatment on fish tissue microbiota, we compared the microbiota of surviving, moribund, and control (untreated) fish. The Shannon diversity was similar between untreated and surviving fish across all tissues but was significantly lower than that observed in moribund fish (Fig. 3A). The nMDS plot shows fin (Fig. 3B) and kidney (Fig. 3C) microbiota of untreated fish tissue microbiota clustered separately from tissue samples following heat treatment (surviving and moribund fish). Pairwise PERMANOVA tests revealed significant Bray-Curtis dissimilarities between fins of untreated versus moribund fish (*P* = 0.006) and between fins of surviving versus moribund fish (*P* = 0.0465). The kidney microbiotas were only significantly different between surviving versus moribund fish (*P* = 0.033).

### *P*/B ratios reveal dysbiosis in fish tissue samples

**Fig 4:**
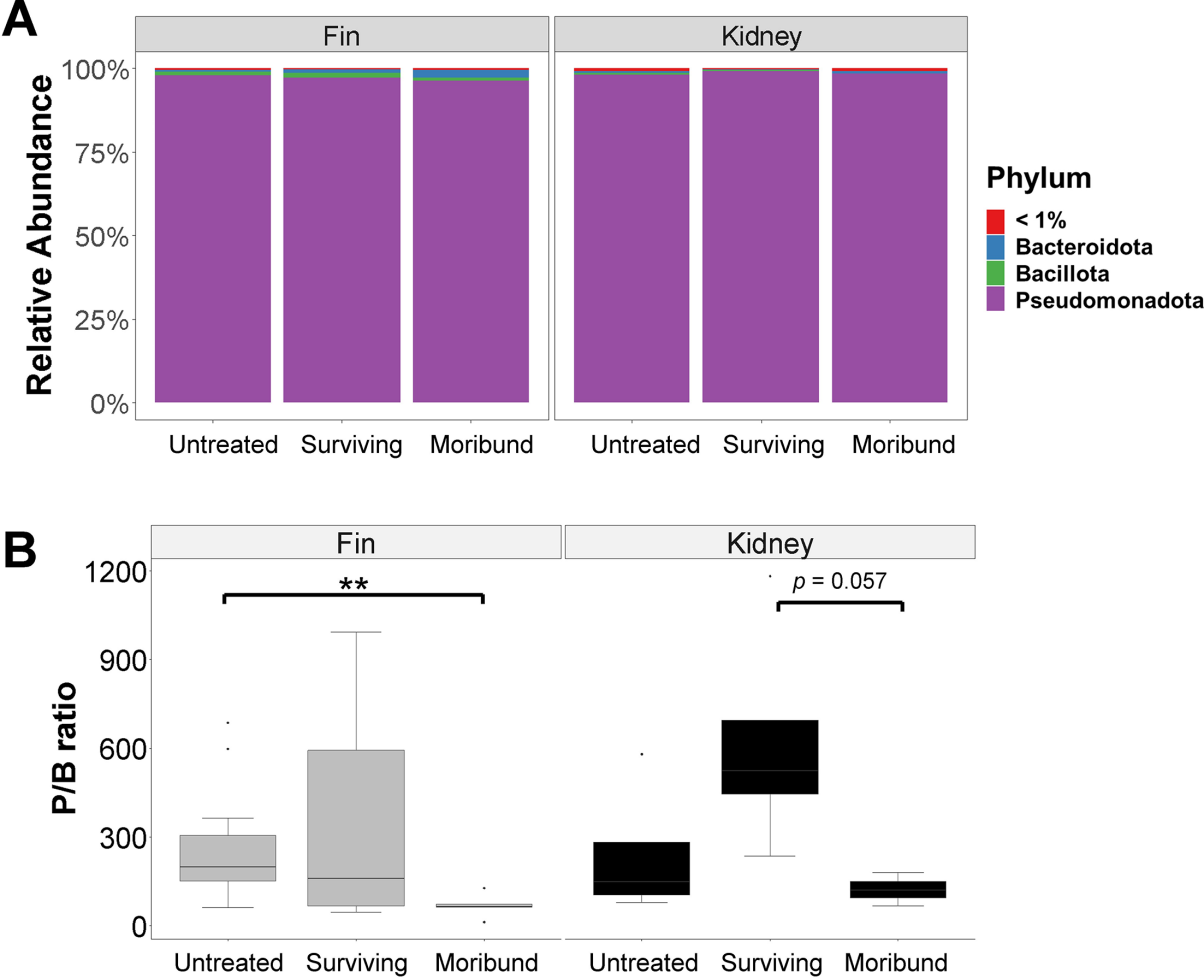
Composition of fin and kidney microbiota in untreated, surviving, and moribund fish in a heat-treated tank. (**A**) Relative abundance of bacterial phyla under heat treatment. Rare taxa in each group, with a relative abundance of less than 1% are indicated as <1%. (**B**) P/B ratio. Differences within the same sample types were tested by Wilcoxon test, with significant pairwise comparisons indicated by *P*-values (*P < 0.05, **P < 0.01, ***P < 0.005) showing based on the connecting compared groups.

Relative abundances of different phyla in tissue samples were analyzed, including both fin and kidney microbiota (Fig. 4A). Fin and kidney of moribund fish had a higher proportion of Bacteroidota and lower proportion of Pseudomonadota compared to untreated and surviving fish (Fig. 4A). However, a significant difference in Pseudomonadota/Bacteroidota (P/B) between moribund and treated or untreated fish is dependent on the fish tissue (Fig. 4B). Nevertheless, the P/B ratio tended to decrease with health status; moribund fish had a lower P/B ratio compared to either untreated or surviving fish (Fig. 4B).

### Potential bacterial drivers of dysbiosis

**Fig 5:**
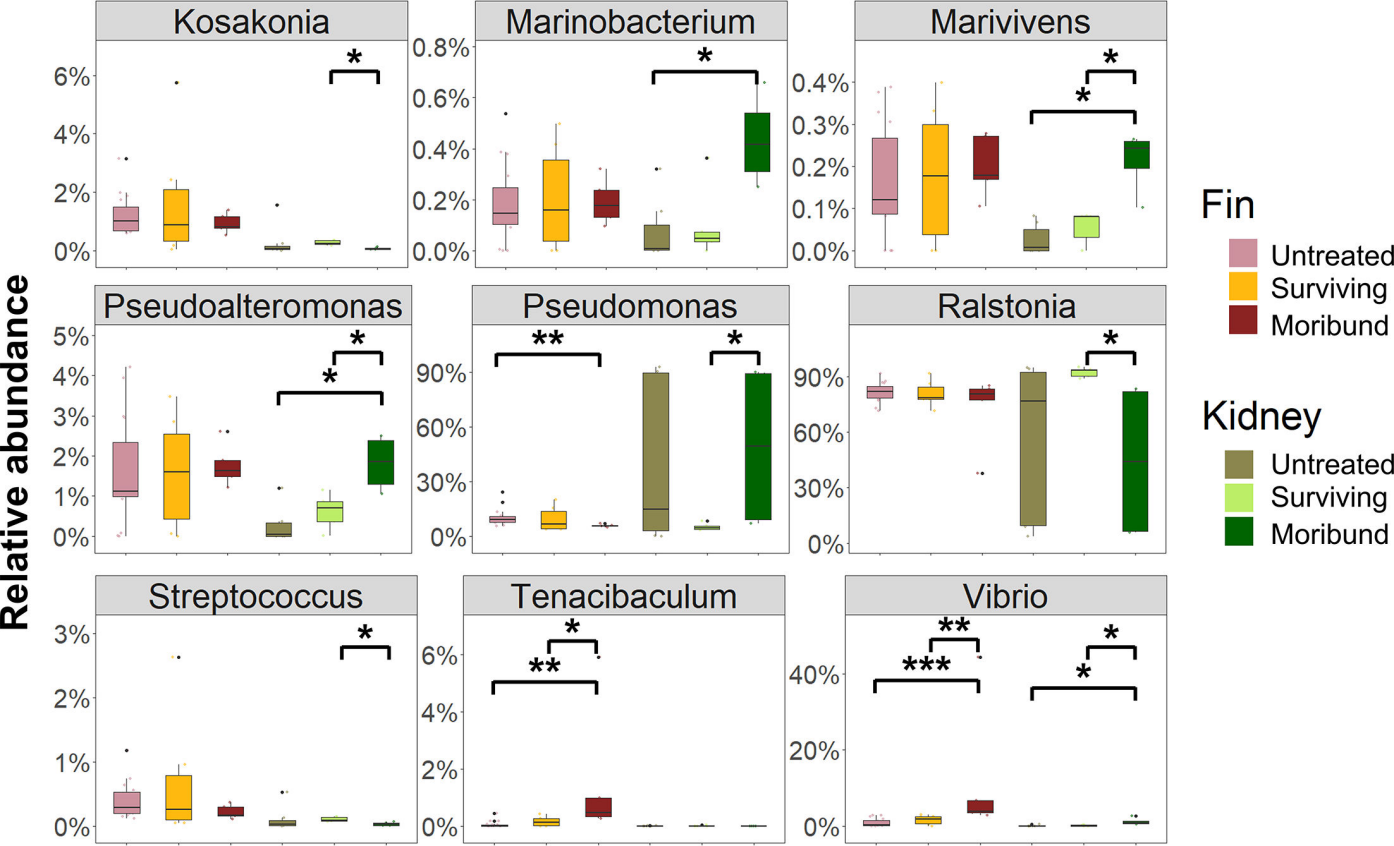
Impact of heat treatment on bacterial genera in fish tissue. Differences within the same sample types were tested by the Wilcoxon test, pairwise comparisons significant *P*-value (*P < 0.05, **P < 0.01, ***P < 0.005) showing based on the connecting compared groups. Untreated: fish collected on non-heat treatment days; surviving: surviving fish collected on heat treatment days; moribund: moribund fish collected on heat treatment days.

The relative abundances of the top genera were compared among tissue samples to assess the effects of temperature on bacterial communities contributing to dysbiosis in tissue microbiotas. Figure 5 presents the number of bacterial genera with significantly different relative abundance between the microbiota of surviving and moribund fish microbiota. The genera, Marinobacterium, Marivivens, Pseudoalteromonas, Pseudomonas, Tenacibaculum, and Vibrio had significantly increased relative abundance in moribund fish. Among these genera, certain species of Pseudomonas, Tenacibaculum, and Vibrio are well-known opportunistic pathogens that can cause infection in fish (50–52). The microbiota of both fin and kidney had a slight decrease in the relative abundance of Streptococcus in moribund fish, another genus that includes some species capable of causing illness in barramundi (53, 54).

### Fish immune genes response to heat treatment

**Fig 6:**
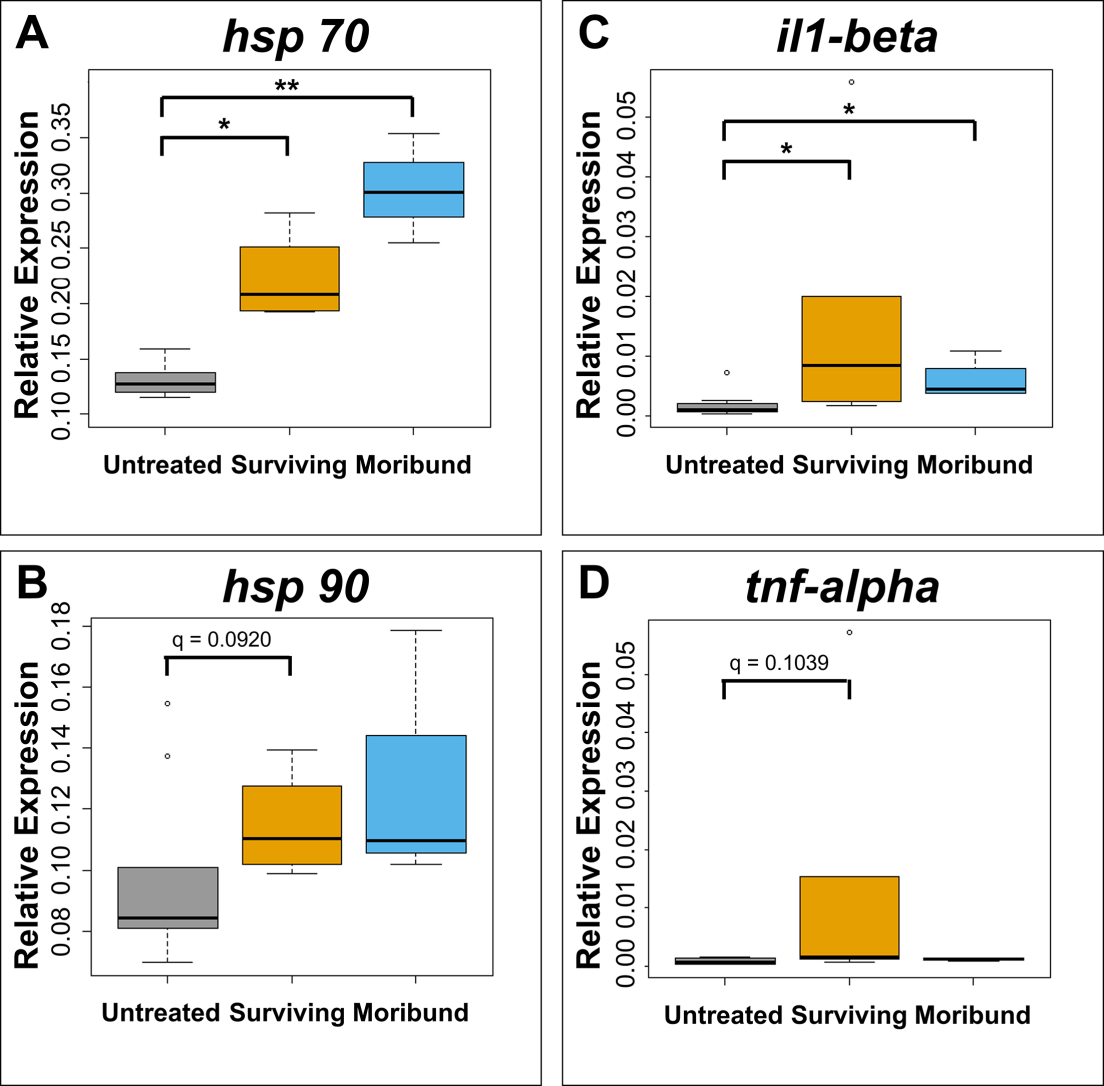
Effects of the heat treatment on host responses. Host genes (**A**) *hsp70*, (**B**) *hsp90,* (**C**) *il1-beta*, and (**D**) tnf-alpha expression were detected in the liver of barramundi using qPCR. Kruskal-Wallis and Dunn’s *post hoc* tests were used to determine differences (* *q* < 0.05, ** *q* < 0.01). Untreated: fish collected on non-heat treatment days; surviving: surviving fish collected on heat treatment days; moribund: moribund fish collected on heat treatment days.

The mRNA levels of heat stress response genes hsp70 and hsp90 increased with rising water temperature (Fig. 6A). Moribund fish had a greater stress reaction, with a higher hsps response in moribund versus surviving fish. The proinflammatory cytokine genes, il1-beta and tnf-alpha, had higher expression levels in surviving versus moribund fish (Fig. 6C and D).

### Quantification of SDDV and*V. harveyi* in fish

**Fig 7:**
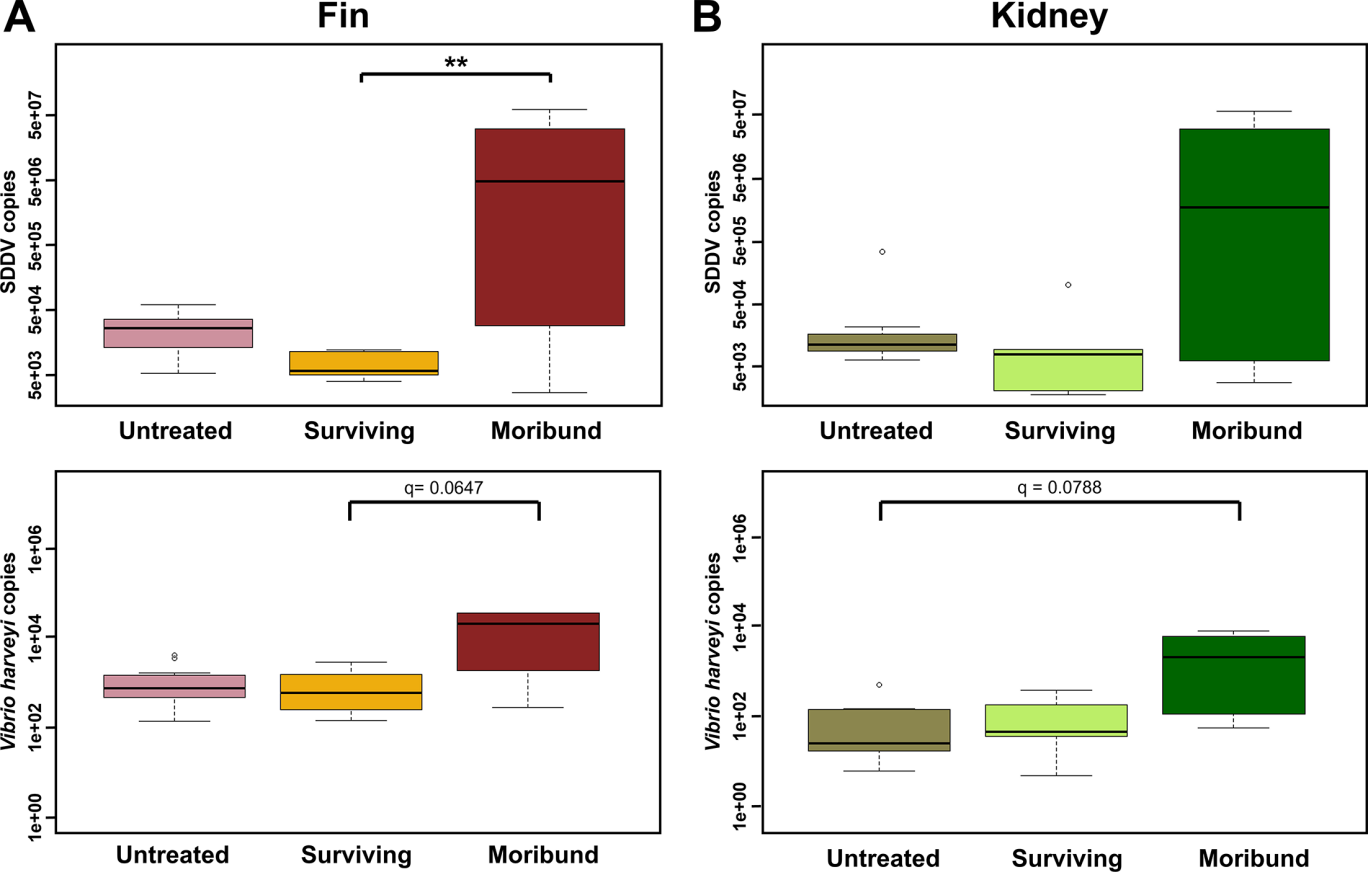
Comparative quantification of SDDV and *V. harveyi* in fish (**A**) fin and (**B**) kidney was detected using qPCR. Kruskal-Wallis and Dunn’s *post hoc* tests were used to determine differences (* *q* < 0.05, ** *q* < 0.01). Untreated: fish collected on non-heat treatment days; surviving: surviving fish collected on heat treatment days; moribund: moribund fish collected on heat treatment days.

 SDDV is a major virus pathogen in farmed barramundi in Singapore, often accompanied by co-infection with *V. harveyi* (44). We used quantitative PCR to detect these two pathogens in fish fin and kidney (Fig. 7). SDDV and *V. harveyi* were found in higher proportions in the tissues of moribund fins compared to untreated and surviving heat-treated fishes. SDDV copies were significantly lower in the fins of surviving fish than untreated fish.

## DISCUSSION

This study investigated the skin microbiota and heat-shock response of barramundi fish subjected to water heat treatment, providing insight into the effects of a common pathogen control method on fish health. Temperature changes can influence both host fitness and microbiota composition, as temperature is a key modulator of bacterial growth. However, few studies have characterized fish skin microbiota under temperature fluctuations, highlighting the importance of this research (15).

We observed a reduction of potential fish pathogens such as *Vibrio* and *Tenacibaculum* spp. in the surviving barramundi following heat treatment. Temperate fishes under heat stress are shown to activate the immune system and reduction of bacteria (55). It has been suggested that the temperature shifts induce the host immune system to control the pathogens (56, 57). Somewhat conflictingly, previous studies found higher water temperatures favored growth of *Vibrio* spp. in salmon gut, particularly during summer (temperature peaked at 18.5°C) compared to the winter (18). However, the temperatures observed in those studies remained within the host’s normal temperature range (10°C–20°C). The heat treatment in this study is about 10°C higher than barramundi’s normal range. There was mortality observed after each heat treatment, demonstrating that drastic temperature changes can cause tissue damage and death (30, 55). Studies in Siberian sturgeon (*Acipenser baerii*) show that water temperatures 8°C higher than the optimal growth of 20°C can harm epidermal cells and skin barrier function by reducing mucus cell numbers and disrupting mucosal microbiota (58). In our study, moribund fish had much higher hsp gene expressions and fish pathogens numbers than surviving fish after heat treatment. Although high water temperatures induced the expression of stress response genes, it may also cause over-response and dysbiosis leading to death. Therefore, determining the threshold of survival temperature for heat treatment is important for different fish species (59).

The elevated expression of *hsp70* and *hsp90* in the liver of fish following heat treatment is consistent with other studies (60). Not only does a temperature change affect immunity but also fish physiological performance and behavior (25, 59). Interestingly, the response to cold stress differs from that of heat stress. Fish innate immune function, such as reduced leukocyte activation and activity of phagocytes, is downmodulated as temperatures decrease (30). Lower temperatures at 15°C were found to inhibit antiviral immune genes interferon and interferon-inducible Mx, but the expression values were generally higher at 28°C which is the normal temperature for zebrafish (*Danio rerio*) development. Whereas some innate immune genes such as *toll-like receptors 3*, il1-beta, and tnf-alpha remained at similar expression levels at both temperatures (58). In contrast, high temperatures (34°C) that are tolerable but exceed the optimum range are mostly linked with the induction of heat-shock proteins and related responses (61). Differential expression of hsp from lower and higher temperature differences has been observed in fish species such as carp and rainbow trout (*Oncorhynchus mykiss*) (60, 62, 63). For example, rainbow trout red blood cells synthesized Hsp70 proteins in response to increased temperature in fish acclimated to 10°C (62).

In the present study, increased levels of il1-beta and tnf-alpha expression in surviving barramundi following heat treatment were observed, although the increase in tnf-alpha expression was not statistically significant, which is congruent with studies of carp fish (64, 65). Concurrent induction of il1-beta and tnf-alpha in response to immune stimulation such as bacterial infection or immune-adjuvants has been reported for rainbow trout, Japanese flounder (*Paralichthys olivaceus*), and grass carp (*Ctenopharyngodon idella*) (66–68). In rainbow trout exposed to 28°C after preconditioning at 19°C for 30–60 min, upregulation of *hsp70*, il1-beta, and tnf-alpha expressions was observed with subsequent heat treatment (69). However, the stress level of pre-conditioned fish was significantly lower than non-pre-conditioned fish. These findings implied that both stress and immune responses can be modulated in fish with repeated heat treatments, and the detailed mechanisms in barramundi need further investigation.

Many current protocols to detect pathogens in farmed fish require killing animals to collect internal organ tissues (e.g., kidney) which may be undesirable for valuable or rare broodstock (70). However, non-lethal sampling for the detection of pathogens, including fin clips and mucus swabs, is growing in practice (71–73). Skin and gill microbiota can be modulated by gut health status and presumably reflect changes in fish health (8). However, limited microbiota research has been conducted on fish internal organs, and it is unclear whether they are fundamentally different from mucosal surfaces such as skin, gills, and gut lining. Microbiota structures of the fish tissue from the present study were distinct from the structure of surrounding water microbiota, which was also the case for previous studies (74, 75). Although some bacteria derived from tissue overlap with those of water microbiota, tissue microbiota often has specific bacterial consortia (16, 49). We propose that skin microbiota may be used to predict microbiota dysbiosis as an indication of infection status. There was a higher proportion of potential opportunistic fish pathogens, such as Vibrio and Tenacibaculum, in the skin and kidney microbiota of moribund fish, with a more significant presence in the skin microbiota. Similar findings were reported in a previous study on farmed diseased barramundi with signs of tenacibaculosis (76). In the present study, there was a change in P/B ratio in surviving and moribund fish. Hence, we propose that the P/B ratio may be a measure of microbiota dysbiosis and the indicator of “unhealthy” farmed barramundi. This is akin to the Bacillota/Bacteroidota (previously known as the Firmicutes/Bacteroidota [F/B]) ratio of human and mammal gut microbiota that have been linked to obesity, diabetes, and ulcerative colitis, among other issues (77). Furthermore, identifying specific bacterial species may be effective indicators for detecting health status, but some may not correspond to the microbiota in another fish species. A previous study also proposed the P/B ratio to predict fish health status (8), consistent with our current observations. We observed that a change in the P/B ratio was more significant in skin samples than in kidney. We inferred that dysbiosis in fish skin communities could provide an early alert regarding opportunistic bacteria. Therefore, monitoring P/B ratio in skin microbiota by non-lethal sampling has the potential to improve fish health monitoring in farmed fish. However, further studies would need to be performed to ascertain the critical P/B ratio at which the fish is “sick” before death. As a result, microbiota detection based on the ratio of P/B instead of F/B ratio could be used in fish.

Despite our findings, there are a few limitations to our study. Our understanding of fish health is largely based on studies performed on temperate fishes, which may differ physiologically from tropical fish. Although only a few genes were tested in this study, the higher levels of cytokine transcripts in surviving fish after heat treatment showed the benefits of heat treatment for fish health. Expanding the study into whole transcriptomics could yield more comprehensive results. Another limitation is the lack of samples for the water column microbiota or chemical quality, which may result in migration or dispersal of microbes on the skin surface as seen in the biofilm studies (78, 79) or reduced dissolved oxygen (80). Water samples could not be collected due to practical and safety concerns on the farm which limited our understanding of the effects of heat treatment on water bacteria community dynamics. While it is evident that temperature changes affected the fish microbiota, the potential interactions with other microbes or the physiochemical properties of the heated water require further investigation. Future studies should include moribund fish from untreated tanks as a comparison to better understand the effects of heat treatment or illness on microbiome alterations.

This study highlights the effect of heat treatment on fish, showing that (i) temperature changes do not impair skin microbiota in surviving fish compared to untreated fish, (ii) significant microbiota changes are expected in moribund fish, (iii) a lower P/B ratio may indicate fish sickness, and (iv) immuno-expression studies support the role of cytokines in the heat-shock response. Future studies should conduct temporal analyzes with larger sample sizes to establish the thresholds for the biomarkers identified.

## Data Availability

Raw sequences are available in NCBI’s Sequence Read Archive (BioProject PRJNA1055435).
